# Peri-Implant Bone Resorption during Healing Abutment Placement: The Effect of a 0.20% Chlorhexidine Gel vs. Placebo—A Randomized Double Blind Controlled Human Study

**DOI:** 10.1155/2018/5326340

**Published:** 2018-10-16

**Authors:** Bruna Sinjari, Gianmaria D'Addazio, Ilaria De Tullio, Tonino Traini, Sergio Caputi

**Affiliations:** University G. d'Annunzio Chieti-Pescara, Department of Medical Oral and Biotechnological Sciences, Via dei Vestini 31, 66100 Chieti (CH), Italy

## Abstract

**Introduction:**

Peri-implant marginal bone loss (MBL) seems to be more pronounced in the first year of loading despite all the studies and changes implemented to reduce it. Among the different causes, the presence of a microgap makes the interface between fixture and abutment colonizable by bacteria, causing an inflammatory response and consequent bone resorption. To reduce this several local antiseptics like chlorhexidine digluconate (CHX) were used after surgical procedures.

**Aim:**

The objective was to radiologically compare the MBL when a 0.20% CHX gel or a placebo gel was applied to the implant-abutment interface during all surgical and prosthetic phases and for a follow-up period up to 12 months.

**Method:**

32 patients (16 for each Group A and B) were enrolled and rehabilitated with a single implant (Cortex classic, Cortex, Shalomi, Israel). During each of the clinical stages a gel containing 0.20% CHX (Plak ®Gel; Polifarma Wellness Srl, Rome, Italy) or a placebo gel (Placebo, Polifarma Wellness Srl, Rome, Italy) was used as indicated by the randomization chart. In order to compare radiographic modification intraoral radiographs was taken. Also, clinical data regarding implant or prosthetic failure and gingival index were recorded. Data were presented as means and standard deviations (SD) and used for the statistical analysis.

**Results:**

All implants showed no bleeding on probing and a very small plaque score at the 1 year of follow-up. MBL was statistically significantly different between the groups in every stage.

**Conclusion:**

Results obtained showed that the use of CHX gel inside the connection significantly reduces MBL during the first year. A rigid disinfection protocol with 0.20% CHX from the time of implant insertion to crown delivery is recommended to reduce host inflammatory response and consequently MBL. This trial is registered with ClinicalTrials.gov Identifier: (Registration Number: NCT03431766).

## 1. Introduction

The implant-supported rehabilitation is, to date, a valid and highly predictable solution for the restoration of missing teeth. The success of this rehabilitation is based on the integration of the implants into the patient's newly formed bone through the osseointegration process. This success has been demonstrated in short and long-term success with 94.6% early success rates and an average success rate of 89.7% even after more than 10 years of function [1–4].

Despite this, the implant-prosthetic treatment has shown different short and long-term complications. Among the early failures are: lack of osseointegration, intra or postoperative infection or loss of the primary stability. Meanwhile, among long-term failure could be found: marginal bone loss (MBL), peri-implantitis, and implant overload [5–9].

Several authors have investigated the causes related to MBL [10, 11]. These studies arise from the need to maintain as much bone as possible around implants obtaining good results in terms of function and aesthetics [10, 11]. During the first year of loading, a certain loss of peri-implant bone is often observed, which tends to decrease in subsequent years [12–14]. Albrektsson and colleagues, in 1986 [13], represent MBL as one of the key factors in the assessment of the implant success rate, describing among the success criteria an MBL up to 1 mm within the first year of implant loading and annual average MBL of 0, 2 mm during the follow-up period.

The possible responsible factors were discussed: implant design, the neck area, implant shoulder surface treatment, surgical trauma, platform switching concept, amount of peri-implant soft tissue, surgical implant placement, residual crestal bone and microgap at the fixture-abutments interface [4, 11, 15, 16].

The latter seems to play a key role in the process of bacterial colonization of the fixture-abutment interface. This area is widely studied in literature in terms of microgap reduction and improvement of the implant-abutment connection. Several authors analyzed the existing microgap describing values between 10 and 135 *μ*m [17, 18]. The different connections commercially available allow in some way to reduce the existing microgap [19–21].

The internal hexagon connection remains in any case the most widespread and simple in terms of clinical procedures. Although new conometric and hybrid connections are able to further reduce the microgap, there are no connections able to completely eliminate this passage area during long-term clinical use [22]. Related to this the study of the fixture-abutment-host interface remains a widely debated chapter in order to reduce or eliminate MBL [15].

The existing microgap could therefore lead to micromovements and bacterial infiltration which results in a peri-implant inflammatory reaction and consequent bone reassembly [17, 18]. Ericsson et al. in 1995 and 1996 observed in an animal study as an infiltrate of inflammatory cells was constantly present at the border between fixture and abutment. In the specimens analyzed, this infiltrate was present both on sites with induced plaque and on cleaned sites. They concluded that this inflammatory infiltrate represented the host's effort to eliminate the bacteria present in the implant system [23, 24].

However, the respective roles of bacterial infection and host response to MBL are not yet well understood. Greater MBL can occur when the host's response is insufficient compared to the infection causing more bone resorption [25].

Over the years the use of rigid surgical and prosthetic protocols has allowed to reduce the microbial load during surgical and postsurgical stage in the peri-implant area. In this sense, the use of local antiseptics such as chlorhexidine (CHX) showed to be a valid support for reducing postsurgical bacterial load [26]. CHX is an agent able to inhibit plaque formation [26, 27] and remains the safest and most effective antimicrobial agent used for the reduction of microorganisms in the oral cavity [28, 29]. CHX is a broad-spectrum antiseptic. It has a pronounced effect on both Gram-negative and Gram-positive bacteria and with bacteriostatic rather than bactericidal activity [27]. The use of CHX-based gels is nowadays part of good clinical practice. In fact, mouth rinses or the application of CHX-based products are used both after simple scaling / root planning and after oral or implant surgery [29]. In recent years, several studies have shown the efficacy of CHX also in the surgical treatment of peri-implantitis. However, its benefits are limited because of its short-term application [30].

The objective of this randomized, double blind, placebo-controlled study was to radiologically compare the MBL when a 0.20% CHX gel or a placebo gel was applied to the implant-abutment interface during all surgical and prosthetic phases and for a follow-up period up to 12 months.

This article was written following the CONSORT statement for improving the quality of RCTs as shown in Figure 6 [31].

## 2. Materials and Methods

### 2.1. Study Design

This prospective, randomized, controlled, double blind clinical trial study was designed according to the Declaration of Helsinki protocol. The allocation ratio was 1:1. The study was approved on 23/07/2015 by the Inter Institutional Ethics Committee of University of Chieti-Pescara, Chieti, Italy; committee report nr:14. All patients gave a written informed consent to the treatment and study recruiting. The trial was registered on clinicaltrials.gov with registration number NCT03431766.

The null hypothesis was that there were no differences between the use of CHX or placebo gel during clinical and prosthetic phase on MBL. The primary outcome was the mean marginal bone loss in single implant-supported restorations. In fact, single unit restoration was chosen to overcome possible problems resulting in patients with multiple implant restorations. MBL was used to estimate the number of patients needed to be randomized.

According to Annibali et al. 2012 [32] a sample size of 15 patients per group was calculated to have at the follow-up a minimum difference of MBL between the two groups of -0.55mm with an expected standard deviation of 0.5 mm. The value of *α* was determined at 0.05 while the power of the test was 0.80. For the calculation, the Pass 3 software was used and specifically the Two-Sample T-Tests taking Equal Variance. A sample size increased by 20% was calculated to avoid patient losses at follow-up which would invalidate the test. So, 18 patients per group were selected.

Meanwhile, the secondary outcomes were as follows:

- Implant failure: implant failure was described as mobility or any infection ordering implant removal.
- Prosthetic failure: prosthetic failure was when it was impossible to place the ceramic restoration or its loss due to fracture or secondary implant failure.
- Gingival index: full mouth plaque score and bleeding on probing score recorded during each stage (4 sides per tooth).
- Any complications and adverse events reported during the first year.

### 2.2. Patient Selection

Forty patients were selected for implant placement. Six were excluded because they did not meet the inclusion criteria. Thirty-four patients (age range 29–75 years; mean age 52,28) without significant medical anamnesis, 14 women and 20 men, all nonsmokers, were recruited as candidates for single implant placement and prosthetic rehabilitation. The patients were enrolled from December 2015 till March 2017 and were treated in the Outpatient Department of Medical, Oral and Biotechnological Sciences of the University “G. d'Annunzio” of Chieti-Pescara, Italy.

The inclusion criteria were as follows:

- Patients between 18 and 75 years
- Good systemic and oral health
- Need of single crown implant-supported restoration
- At least six months of healing after tooth extraction
- Cortical bone thickness > 5 mm measured by means of a cone beam computed tomography (CBCT)
- Adequate dimension of the attached gingiva (>2 mm) or keratinized tissue at the site selected

The exclusion criteria were as follows:

- Poor oral hygiene
- Active periodontal disease or other oral disorders
- Insufficient bone thickness for implant insertion
- Bone augmentation procedures
- Immediate loading protocols
- Uncontrolled diabetes mellitus
- Immune diseases
- Smoking
- Bruxism.

### 2.3. Randomization

Patients were randomly divided into Group A (control) and Group B (test) as indicated by the randomization chart. The randomization was obtained using computer generated random numbers, centralized with sequentially sealed opaque envelopes provided by the study adviser.

The investigation team was composed by a principal investigator who designed the study and made the randomization, the surgeon who performed first and second stage surgery, examiners who performed the radiological analysis, dentist who performed the prosthetic procedure, and the statistician. Patients were informed of all the study procedures but blinded to the different gel used in the study.

The surgeon opened the sealed envelope containing the randomized group only after having inserted the implant.

### 2.4. Surgical and Prosthetic Treatment

**Figure 1:**
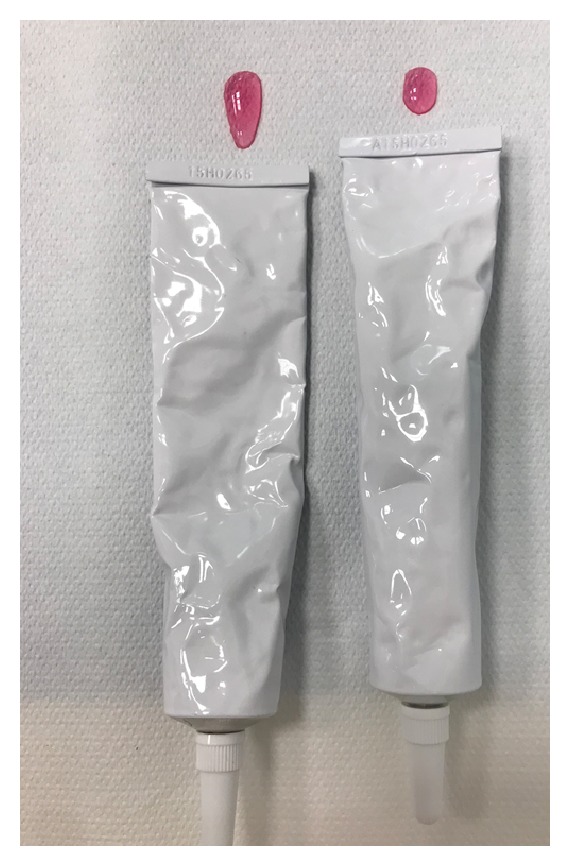
The two gels used during every stage of the study. Gels were perfectly alike in packaging, colour, and smell.

During the first evaluation, all subjects were clinically examined. Radiographs and gingival index, such as, plaque and bleeding scores, were carried out for diagnostic evaluation; then the patients were scheduled for surgery procedures. All implants (Cortex classic, Cortex, Shalomi, Israel) were inserted (T0) by two skilled operators who followed a two-stage protocol and placed them according to the manufacturer's instructions. Before surgery all the patients were subject to mouth rinse of chlorhexidine digluconate solution 0.2% for 2 minutes to reduce the bacterial load and local anesthesia was given with Articaine® (Ubistesin 4% - Espe Dental AG Seefeld, Germany) associated with epinephrine (1:100.000). Eventually all patients were rehabilitated with a single implant-supported crown. During all stages either a gel containing 0.20% chx (Plak ®Gel; Polifarma Wellness Srl, Rome, Italy) or a placebo gel (Placebo, Polifarma Wellness Srl, Rome, Italy) was used. The two gels were perfectly alike in packaging, colour, and smell and nobody knew the exact location of placebo or test gel which was revealed, only after data collection was performed, by the person who prepared them. (Figure 1) All implants achieved primary stability with a mean insertion torque of 39Ncm.

A or B gel was placed on internal connection abutments and then a cover screw was inserted. Finally the site was sutured with nonabsorbable sutures. Cone Beam Computed Tomography evaluation (CBCT) (Vatech Ipax 3D PCH-6500, Fort Lee, NJ, USA) was performed for both preoperative (to evaluate residual bone) and postsurgical implant placement. All patients received antibiotic therapy, 2g/day for 6 days (Augmentin®; Glaxo-Smithkline Beecham, Brentford, UK).

The postoperative pain was controlled with NSAIDs, and oral hygiene instructions were given. Both gels were given to the patients, according to the protocol and applied 2 times/day until suture removal after 7 days. Implants were allowed to a submerged heal of 8 weeks. Eight weeks later, implant stability was assessed by percussion test, the second surgical stage was performed, and a healing abutment was placed (T1). Using an open tray technique an impression was made and an acrylic provisional restoration was positioned (T2, 16 weeks after surgery). Finally, porcelain fused to metal restoration was inserted 18-20 weeks after implant placement. A follow- up period of 12 months was established.

### 2.5. Data Handling and Radiographic Analysis of Marginal Bone Level Changes

Implant success was evaluated according to the clinical and radiographic criteria of Papaspyridakos et al. 2012 [33]: (1) absence of implant mobility; (2) absence of pain; (3) absence of recurrent peri-implant infection; and (4) absence of a continuous radiolucency around the implant. Data were collected to the specific patient's CRFs.

The peri-implant and gingival index were recorded. Specifically, plaque score (PS) and bleeding on probing (BOP) were evaluated and recorded on four surfaces on each tooth at every study time points. Radiographs and clinical photographs were also recorded during every stage: visit, first implant surgery, second surgical stage, at provisional and permanent restoration positioning, and at follow-up visits (at 6 and 12 months). Also during each visit possible adverse events were recorded. In any case placebo or CHX gel was used during every implant-abutment connection. In order to compare radiographic modification of the peri-implant marginal bone, intraoral radiograph, applying the parallel ray technique, was taken immediately after implant insertion. To measure the crestal bone remodeling, intraoral analogic Rx was taken during each stage and processed on a digital software because the method was considered of high precision for scientific evaluations with a precision less than 0.1 mm [34, 35]. The mean value between mesial and distal region was used as the primary outcome measure for this study.

In order to obtain a highly reproducible and faithful image, the commercially available Rinn film holders, used for intraoral radiographs applying the parallel X-ray technique, were customized using a silicone key for the exact reposition in every subject. Also, during the first radiography kilovolts, milliampere, and seconds were recorded and used every time to obtain the same radiographs. Radiographs were repeated at the implant placement (T0), at 8 weeks during second surgical stage (T1), at 12 weeks during provisional delivery (T2), at 14 weeks during restoration placement (T3), and at 12 months of follow-up (T4).

**Figure 2:**
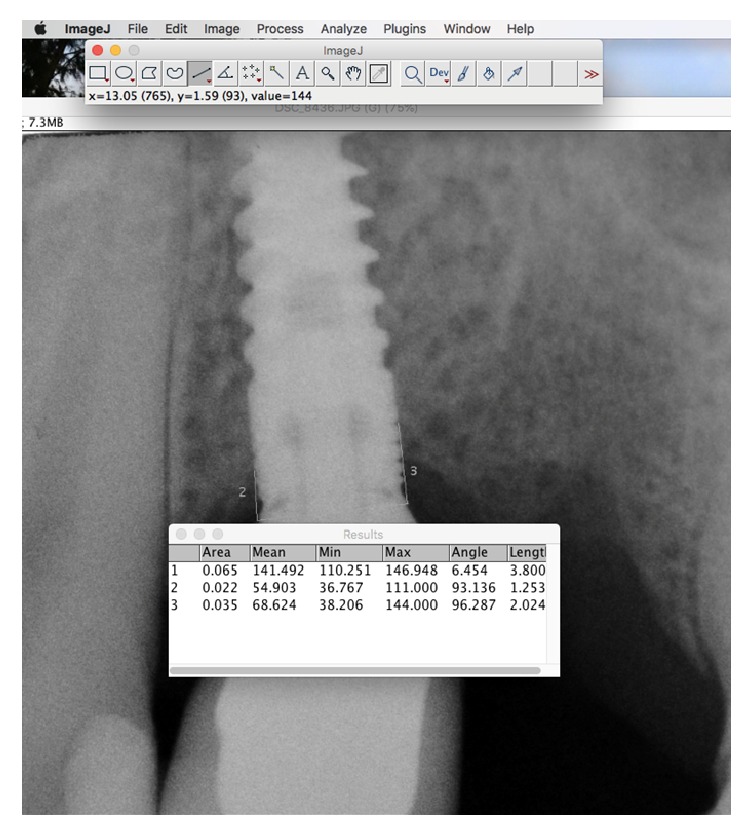
A computer-assisted calibration was performed and linear measurements of MBL were Also it was measured the known implants length and diameter to guarantee a correct measurement even if the implant was slightly angulated on the radiograph.

In every radiograph distance from the top of the fixture and the mesial and distal crestal bone level measured to the first bone to implant contact were recorded. The mean value between mesial and distal region was calculated for the data analysis. Also the known implants length and diameter were measured to guarantee a correct measurement even if the implant was slightly angulated on the radiograph. (Figure 2) Based on this ratio a computer-assisted calibration was performed and linear measurements of MBL were taken using ImageJ 1.48 v.; Bethesda, MD

### 2.6. Statistical Analysis

Statistical tests were conducted with SPSS (IBM Corp., Armonk, NY, USA) and Excel (Microsoft, Redmond, WA, USA) software. The statistical tests to be used were predetermined by the study protocol. Patients were considered for statistical evaluation. Data was presented by means and standard deviations (SD). Analysis of variance (Student's t-test) was used to evaluate differences between groups. Tukey test at 5 different time points considered into the study was used to evaluate difference among groups. Significance was set to p=0.05.

## 3. Results

**Figure 3:**
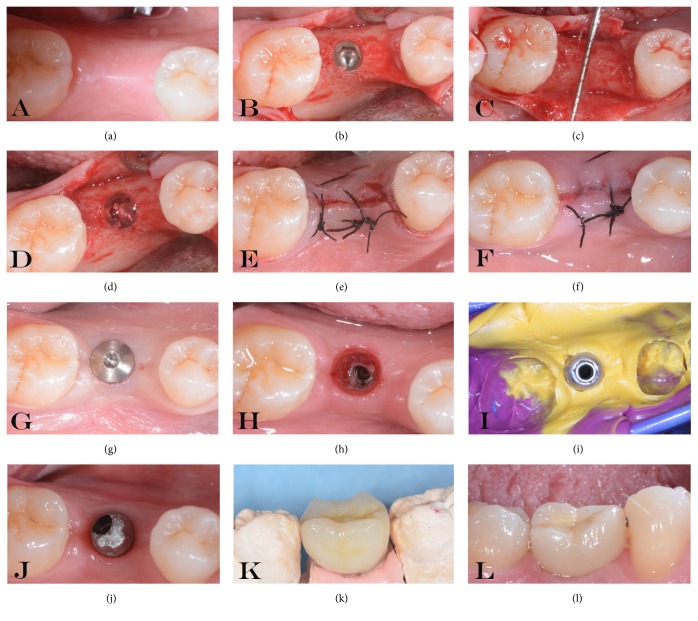
Explanatory images of all the treatments performed (a-l). (a) Residual crest. (b) Implant insertion. (c) Measurement of residual bone. (d) Gel inserted into the fixture. (e) Suture positioning. (f) Suture removal. (g) Second surgical stage. (h) A minimal inflammatory response was present below the healing screw. (i) Impression. (j) Abutment positioning and temporary crown delivery. (k-l) Definitive ceramic restoration delivery.

Throughout the study, 40 patients were screened for inclusion and exclusion criteria. 34 patients were enrolled for single implant-supported restoration. Six patients were rejected for not meeting the inclusion criteria. Furthermore, two patients were excluded after randomization due to poor oral hygiene at the surgery appointment. A total of 16 patients were included in Group A (control group with placebo gel). Meanwhile, 16 patients were included in Group B (chlorhexidine gel) as shown in Table 1. All implants placed were successfully integrated, with no clinical signs of peri-implant infection or mucositis at 1 year of follow-up. So, no dropouts occurred during the study (survival rate of 100% at 12 months of osseointegration). The prosthetic restorations were well integrated and no prosthetic complications were recorded (no unscrewing, ceramic chips, or fractures). In Figure 3 it was possible to observe an explanatory case of all the treatments performed. Specifically, the patient was within Group A. A minimal inflammatory response was present below the healing screw (letter H). The patients included were all under strict hygienic periodontal control. All registered indices (plaque score and bleeding score) demonstrated that they, in both groups, were below 25% during all phases of treatment. (Table 2) A slight increase (mean 23,45% in Group A and 23,28% in Group B) was recorded at the annual control visit, but without statistically significant differences.

**Figure 4:**
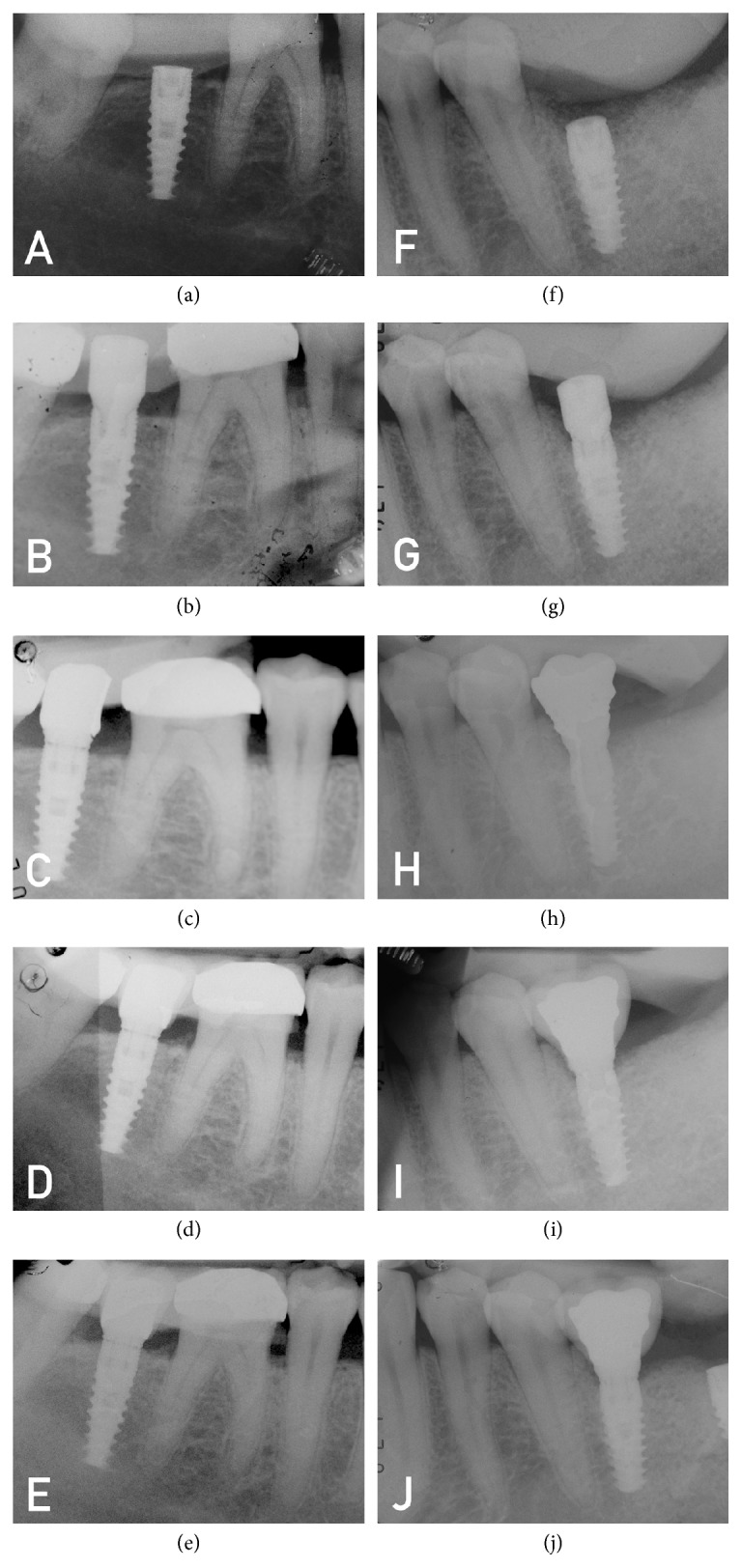
Radiographs from the two groups. (a-e) Patient from Group B (test group). A minimal bone gain was present at the second surgical stage. (f-j) Patient from Group A (control group).

**Figure 5:**
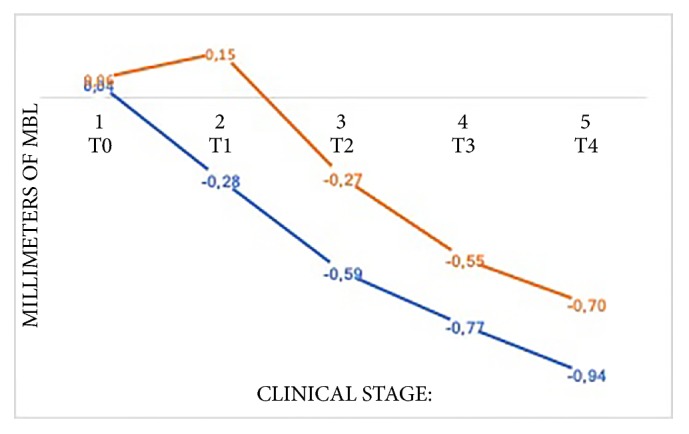
Graphical representation of MBL during every surgical and prosthetic stage.

**Figure 6:**
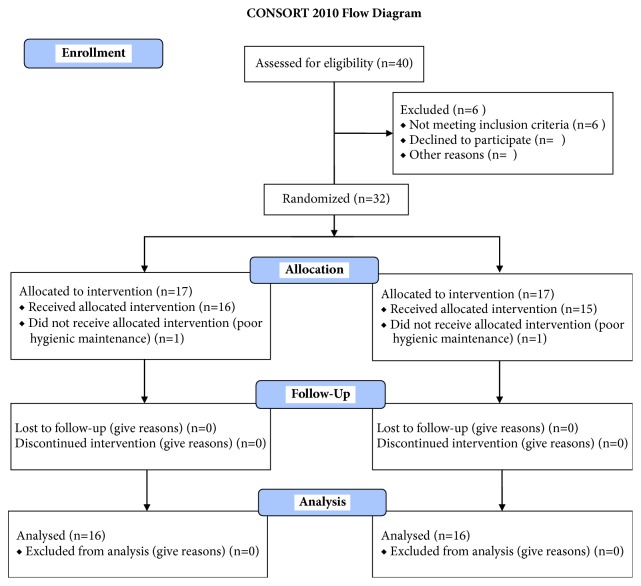
CONSORT 2010 Flow Diagram. Flow diagram of the progress through the phases of a parallel randomized trial of two groups.

At T0 (implant placement), the mean value was 0.04 ± 0.30 mm for Group A and 0.06 ± 0.22 mm for Group B, without significant statistical difference. Analysis of data showed different MBL into the two groups during the different stages as shown in Table 3. (Figures 4 and 5) Specifically, at T1 (second surgical stage) the mean MBL values were -0.28 ± 0.40 mm in Group A and 0.15 ± 0.25 mm in Group B. A small mean bone gain was found in Group B. Statistically significant differences were present between the two groups. The mean bone loss for the groups at the time points T2 (provisional delivery) and T3 (definitive delivery) was -0.59 ± 0,35 mm and -0.77 ± 0.30 mm in Group A and -0.27 ± 0.18 mm and -0.55 ± 0.17 mm in Group B, respectively. At 12-month follow-up examination, T4, the MBL was -0,94 ± 0.33 mm in Group A and -0.70 ± 0.16 mm in Group B. Most of the MBL occurred in both groups from the time of second surgical stage to 12 months of follow-up. Statistical analysis showed statistically significant differences between the two groups during each time point. Statistically significant differences were also present at the time of second surgical stage as shown in Table 4.

## 4. Discussion

The aim of the study was to compare the effect of a 0,20% CHX gel or placebo used during all the restoration phases on the MBL rate around implant-supported rehabilitation.

To reduce bias on both groups, only the gel used during connection decontamination was changed; the same type of implant design, surface treatment, and type of connection was used [36]. Meanwhile, the type of gel was different: 0,20% CHX gel on Group A and placebo gel on Group B, respectively.

To the authors' best knowledge this is the first study which investigates the effects of 0,20% CHX gel during the early stage of healing abutment connection (T1) and provisional prosthetic delivery (T2) on MBL. Until now, there was no scientific evidence to explain the mechanisms related to MBL around implants related to the use of CHX. Specifically, MBL was used as a measure of treatment success; a prognostic factor leads to capture the effect of treatment on the clinical endpoint but not to directly measure the main clinical benefit of the intervention [37].

Several authors have investigated the MBL around the implants demonstrating how most of the bone reabsorption occurs during the first year of loading [38–40].

According to Bateli et al., the possibility of preserving MBL requires a multifactorial approach [41]. Factors like surgical trauma, biologic width establishment, absence of passive fit of the super- structures, implant-abutment microgap, and occlusal overloading have been studied [7, 9, 11, 16]. In our study the influence of implant design, platform switching, neck roughness, and different connection types was excluded using the exact same type of implants in both groups analyzed. Moreover, all the implants were inserted using the same surgical technique, by the same operator, obtaining a mean insertion torque of 39 Ncm.

Regarding the MBL the overall mean values of the two groups were 0.05 ± 0,26 mm at the baseline. Following the protocol, the implants were placed all at bone level and all by the same operator to receive high accuracy. Considering this, MBL at 12 months was -0,94 ± 0.33 mm and -0.70 ± 0.16 mm for Group A and Group B, respectively. Although there was a statistically significant difference between the groups, the values in both were in agreement with other authors [38, 39, 42, 43]. On the other hand, the major part of MBL occurred at the temporary crowns delivery. It was important to underline that there was a statistically significant difference also at T2 (second surgical stage) between the two groups. A possible interpretation of these data, considering the observed inclusion criteria, the correct surgical technique, and postoperative control, could be linked to the effect of CHX positioned on the internal part of the implant fixture. The latter may have reduced the microbial load at the implant bone interface. So, it could reduce the host's response due to the inflammatory infiltrate and early peri-implant bone resorption. According to Traini et al. [43], an important percentage of MBL occurs after the healing abutment connection, where the possibility of microbial load through the abutment connection placement could be considered a cause of bone loss during the healing period. In this sense, bacterial colonization of fixture-abutment interface could play a key role in this process.

Other authors have shown how different connections can reduce the existing microgap, improving fixture-abutment seal and reducing micromovements [18, 19, 25]. However, it was shown that, also in conometric connections, completely filling this space was almost impossible [22]. In the present study, it has been decided to use an internal hexagonal connection as it is widely described in the literature in terms of function and long-term stability [4, 10, 36]. Despite this, the existing microgap described in any type of connection makes necessary a strict disinfection protocol of the internal portion of the fixture to reduce bacterial colonization.

Short-term studies [44, 45] showed how cemented fixture-abutment connections could eliminate the presence of the microgap, filled by cement. However, to the best of the authors' knowledge there are no long-term studies that guarantee these results. On the other hand, cement retained abutments may cause peri-implantitis due to the presence of residual cement under the abutment connection [46].

Despite the presence of several studies concerning MBL, no one has specified the time course of this reabsorption [47–49]. In an animal study, hermann et al. [50] demonstrated how bone loss events occurred after the second surgical stage. Several authors attributed this process exclusively to biological width formation [12, 15, 51]. Many studies have clarified the role of platform switching and the formation of biological width. It has been widely demonstrated that a configuration with mismatching of the abutment leads to a lower bone resorption by introducing a horizontal component of the biological width [51]. In this way the peri-implant inflammatory area was translated internally, making a minor MBL and greater prosthetic and soft tissue aesthetic results. Canullo et al. [52] demonstrated the importance of platform switching in immediate loading restorations in a long follow-up period. In a 10-year study, 22 patients were rehabilitated with an immediate loading single implant, half with platform switching and half with a standard solution. With regard to MBL, statistically better results were obtained in the test group, even 10 years of function. This demonstrates the important role of platform switching and the formation of biological width in peri-implant bone maintenance. Moreover, good results were obtained in the clinical evaluation of the aesthetic aspect and the presence of the interimplant papilla, where better results were present in the test group [52]. Although in agreement with these hypotheses, all our completed rehabilitation was realized without platform switching. Platform matched solution was chosen in order to avoid interference with our findings. However our results were comparable with platform switching related studies [52, 53].

Likewise, in the presented results, the reduction of the microbial load has been transformed into a reduction of the inflammatory response with results in terms of MBL comparable to platform switching rehabilitation. Anyway, longer follow-up would be necessary to verify the efficacy of CHX in this rehabilitation. Romanos et al. [54] studied how the use of CHX effectively reduced the microbiological load into the fixture-abutment connections. However, these differences were not statistically significant. They studied the colonization into two different types of connections, internal hexagon and conometric, in one month of follow-up. Their results showed that, in any case, a bacterial load was always present inside the connection, even if with very encouraging results after the use of CHX. It is important to underline that, during the observation period, all the samples were decontaminated with chlorhexidine. For this reason, there were no control groups to evaluate the status of the connection with and without chlorhexidine use [54]. In contrast, our 12-month follow-up demonstrates how, despite the permeability of the interface, the use of CHX gel significantly improves the response of the peri-implant tissue with a significant reduction of MBL. Moreover, in 2008, Paolantonio et al. [55] investigated the role of CHX in decontamination of the internal portion of the internal hexagon implant connection. At six months of loading the abutments were removed to assess the presence, through real-time PCR, of bacteria in test (treated with CHX) and control (untreated) groups. In both groups bacteria were present, demonstrating how this type of connection cannot guarantee a good seal between the components. In any case, in the test group the presence of bacteria was statistically lower than the control group, demonstrating the effectiveness of the decontamination protocol. However, clinical and radiological evaluations were not performed [55]. The present study has a double follow-up compared to the previous one and evaluates the efficacy in terms of MBL and reliable clinical and radiological index of the health status of implant-supported rehabilitation. Different studies tested the antimicrobial capacity of CHX in the decontamination of implant surfaces affected by peri-implantitis [24, 30]. This treatment was carried out for short periods of time (during peri-implantitis treatment). Therefore it was not possible to make any considerations regarding the continuous use and the effects of CXH over time. A clinical study about the decontamination of the implant surface with 0.12% CHX during the surgical treatment of peri-implantitis determined a reduction of the anaerobic microbial load without, however, improving the clinical success [56]. This could be due to the surgical technique rather than the decontamination method [57]. In addition, CHX has bacterial capacity, but it is not able to remove the acquired film on the implant surface [58]. In addition, the 0.12% CHX concentration may be too low to have sufficient effects to reduce the microbial load [56].

In any case, the study was based on a number of patients; even if the number is statistically acceptable, it is still limited. Therefore, further investigations with more relevant sample sizes are needed. However, the patients are still under strict control and results from longer follow-up periods (5 and 10 years) should be investigated. Therefore, albeit with due caution, the CHX gel formulation used allowed us to obtain encouraging results, considering the rigid selection of patients, the protocol, the randomization technique, and the observation performed (double blind). In any case, further investigation may be necessary to have a more complete draft, like a microbiological profile or a peri-implant soft tissue analysis to assess the inflammatory status of soft tissues. It could also be useful to perform this investigation in different connection types and evaluate its effectiveness following strict decontamination protocols.

## 5. Conclusion

The results obtained from this randomized double blinded human-controlled study showed that the use of 0,20% CHX gel inside the connection during all surgical and prosthetic phases significantly reduces peri-implant crestal bone loss during the first year. However, the existing space between fixture and abutments remains a crucial area in bacterial colonization as a starting point for the MBL around the fixture. A rigid disinfection protocol with 0.20% CHX from the time of implant insertion to crown delivery is strongly recommended to reduce host inflammatory response and consequently MBL.

## Figures and Tables

**Table 1:** Patient characteristics at study baseline.

**Patient id**	**Sex**	**Age**	**Group**	**Implant Site**	**Implant Dimension**	**Bone Density**	**Final Torque NCM**
1	F	57	B	3.6	3.8x11,5	Normal	35
2	F	32	A	1.6	4.2x11,5	Poor	40
4	M	35	B	3.6	3.8x11,5	Normal	30
5	F	45	A	1.4	3.8x11,5	Poor	35
6	M	49	B	1.6	4.2x10	Normal	35
7	F	51	A	4.6	4.2x11,5	Dense	35
8	M	67	B	4.6	4.2x10	Normal	45
10	F	38	B	3.6	3.8x11,5	Dense	40
11	F	29	A	1.6	4.2x10	Poor	45
12	M	70	B	2.4	3.8x11,5	Normal	35
13	F	68	B	3.6	4.2x11,5	Normal	40
14	F	56	A	4.7	3.8x10	Normal	45
15	M	75	B	3.6	3.8x11,5	Dense	50
16	M	39	B	4.6	4.2x10	Dense	35
17	F	40	A	3.6	3.8x10	Dense	30
18	M	41	B	1.5	3.8x11,5	Poor	35
19	F	50	B	4.6	4.2x11,5	Normal	30
20	M	67	A	3.6	3.8x11,5	normal	35
21	M	71	A	4.6	4.2x11,5	Normal	40
22	F	70	B	2.4	3.8x11,5	Normal	45
23	M	40	A	4.6	3.8x11,5	Dense	40
24	F	59	A	3.5	4.2x11,5	Normal	35
25	M	63	A	2.2	3.8x11,5	Dense	45
26	M	68	B	4.5	4.2x10	Normal	40
27	M	56	B	3.7	3.8x11,5	Normal	35
28	F	59	B	2.5	4.2x10	Poor	45
29	F	44	A	3.6	4.2x10	Dense	40
30	M	43	A	3.7	3.8x11,5	Dense	40
31	M	39	B	2.6	4.2x11,5	Poor	35
32	M	35	A	2.4	3.8x11,5	Normal	45
33	F	56	A	3.6	3.8x11,5	Dense	50
34	M	61	A	4.6	4.2x11,5	Dense	40

**Table 2:** Average percentage for the different groups of plaque score (PS) and bleeding scores (BS) recorded during all phases of the study.

	**T0 A**	**T0 B**	**T1 A**	**T1 B**	**T2 A**	**T2 B**	**T3 A**	**T3 B**	**T4 A**	**T4 B**
PS	17.45	17.32	19.34	18.45	17.34	18.32	20.32	19.46	23.45	23.28
BS	9.2	8.15	11.32	12.21	13.45	13.78	15.21	16.32	17.25	18.5

**Table 3:** Table shows different MBL into the two groups during the different stages.

**ID PAT**	**SITE**	**GROUP**	**T0**	**T1**	**T2**	**T3**	**T4**
2	1.6	A	0.22	-0.06	-0.49	-0.73	-1.51
5	1.4	A	0.00	-0.38	-0.55	-0.82	-1.07
7	4.6	A	0.49	0.12	-0.20	-0.84	-0.95
11	1.6	A	-0.62	-1.53	-1.75	-1.76	-1.84
14	4.7	A	-0.39	-0.92	-0.89	-0.65	-0.80
17	3.6	A	-0.06	-0.38	-0.43	-0.68	-0.73
20	3.6	A	0.06	0.00	-0.44	-0.60	-0.63
21	4.6	A	0.16	-0.29	-0.53	-0.73	-0.94
23	4.6	A	-0.13	-0.20	-0.74	-1.01	-1.05
24	3.5	A	-0.06	-0.20	-0.44	-0.59	-0.64
25	2.2	A	0.60	-0.06	-0.46	-0.71	-0.77
29	3.6	A	-0.11	-0.15	-0.78	-0.94	-1.06
30	3.7	A	0.05	-0.09	-0.25	-0.38	-0.58
32	2.4	A	0.35	-0.05	-0.52	-0.79	-0.90
33	3.6	A	0.21	-0.05	-0.40	-0.58	-0.74
34	4.6	A	-0.08	-0.21	-0.58	-0.61	-0.80
		**mean **	**0.04**	**-0.28**	**-0.59**	**-0.77**	**-0.94**
		**SD**	**0.30**	**0.41**	**0.36**	**0.30**	**0.33**

1	3.6	B	0.57	0.62	0.00	-0.47	-0.55
4	3.6	B	-0.17	0.03	-0.50	-0.71	-0.81
6	1.6	B	0.10	0.22	-0.05	-0.41	-0.93
8	4.6	B	0.20	0.21	-0.48	-0.74	-0.79
10	3.6	B	-0.04	0.00	-0.38	-0.53	-0.61
12	2.4	B	0.00	0.10	-0.35	-0.55	-0.71
13	3.6	B	-0.02	0.15	-0.37	-0.67	-0.89
15	3.6	B	0.16	0.39	-0.08	-0.61	-0.74
16	4.6	B	0.12	0.59	-0.23	-0.49	-0.61
18	1.5	B	0.00	-0.36	-0.57	-0.69	-0.77
19	4.6	B	0.34	0.24	0.00	-0.16	-0.31
22	2.4	B	0.29	0.28	-0.29	-0.88	-0.95
26	4.5	B	-0.35	-0.08	-0.15	-0.41	-0.66
27	3.7	B	0.00	-0.05	-0.21	-0.54	-0.59
28	2.5	B	0.06	0.18	-0.30	-0.37	-0.57
31	2.6	B	-0.26	-0.08	-0.46	-0.56	-0.70
		**mean **	**0.06**	**0.15**	**-0.27**	**-0.55**	**-0.70**
		**SD**	**0.23**	**0.25**	**0.18**	**0.17**	**0.16**

**Table 4:** Statistical analysis compared the different MBL between the two groups in every single stage.

**T0**	0,0435625	T-ratio	-0,220803459	
**t0 b**	0,0621875	DF	15	
Mean difference	-0,018625	Prob > |t|		0,8282
Std. error	0,084351034	Prob > t		0,5859
Upper 95%	0,161164972	Prob < t		0,4141
Lower%	0,198414972			
N	16			
Correlation	0,211215722			

**T1**	-0,2768125	T-ratio	-3,690326917	
**T1 b**	0,15328125	DF	15	
Mean difference	-0,43009375	Prob > |t|		0,0022
Std. error	0,116546246	Prob > t		0,9989
Upper 95%	-0,181681306	Prob < t		0,0011
Lower%	-0,678506194			
N	16			
Correlation	0,060006213			

**T2**	-0,62671875	T-ratio	-4,200440951	
**T2 b**	-0,2736875	DF	15	
Mean difference	-0,35303125	Prob > |t|		0,0008
Std. error	0,084046236	Prob > t		0,9996
Upper 95%	-0,173890939	Prob < t		0,0004
Lower%	-0,532171561			
N	16			
Correlation	0,394342209			

**T3**	-0,846	T-ratio	-3,483100821	
**T3 b**	-0,54834375	DF	15	
Mean difference	-0,29765625	Prob > |t|		0,0033
Std. error	0,085457259	Prob > t		0,9983
Upper 95%	-0,115508414	Prob < t		0,0017
Lower%	-0,479804086			
N	16			
Correlation	0,407963714			

**T4**	-1,033375	T-ratio	-2,787533871	
**T4 b**	-0,69665625	DF	15	
Mean difference	-0,33671875	Prob > |t|		0,0138
Std. error	0,120794496	Prob > t		0,9931
Upper 95%	-0,079251376	Prob < t		0,0069
Lower%	-0,594186124			
N	16			
Correlation	0,103410841			

## Data Availability

The data used to support the findings of this study are included within the article.
